# Interplay of body mass index and metabolic syndrome: association with physiological age from midlife to late-life

**DOI:** 10.1007/s11357-023-01032-9

**Published:** 2023-12-16

**Authors:** Peggy Ler, Alexander Ploner, Deborah Finkel, Chandra A. Reynolds, Yiqiang Zhan, Juulia Jylhävä, Anna K. Dahl Aslan, Ida K. Karlsson

**Affiliations:** 1 Department of Medical Epidemiology and Biostatistics, Karolinska Institutet, Nobels väg 12A, Solna, 171 65 Stockholm, Sweden; 2 Center for Economic and Social Research, University of Southern California, Los Angeles, California USA; 3 Institute of Gerontology, Jönköping University, Jönköping, Sweden; 4 Institute for Behavioral Genetics, University of Colorado Boulder, Boulder, Colorado USA; 5 School of Public Health, Sun Yat-Sen University, Shenzhen Campus, Shenzhen, Guandong China; 6 Faculty of Social Sciences, Unit of Health Sciences and Gerontology Research Center, University of Tampere, Tampere, Finland; 7 School of Health Sciences, University of Skövde, Skövde, Sweden

**Keywords:** Biological age, Frailty index, Metabolic syndrome, Metabolic health, Obesity

## Abstract

Obesity and metabolic syndrome (MetS) share common pathophysiological characteristics with aging. To better understand their interplay, we examined how body mass index (BMI) and MetS jointly associate with physiological age, and if the associations changed from midlife to late-life. We used longitudinal data from 1,825 Swedish twins. Physiological age was measured as frailty index (FI) and functional aging index (FAI) and modeled independently in linear mixed-effects models adjusted for chronological age, sex, education, and smoking. We assessed curvilinear associations of BMI and chronological age with physiological age, and interactions between BMI, MetS, and chronological age. We found a significant three-way interaction between BMI, MetS, and chronological age on FI (p-interaction = 0·006), not FAI. Consequently, we stratified FI analyses by age: < 65, 65–85, and ≥ 85 years, and modeled FAI across ages. Except for FI at ages ≥ 85, BMI had U-shaped associations with FI and FAI, where BMI around 26-28 kg/m^2^ was associated with the lowest physiological age. MetS was associated with higher FI and FAI, except for FI at ages < 65, and modified the BMI-FI association at ages 65–85 (p-interaction = 0·02), whereby the association between higher BMI levels and FI was stronger in individuals with MetS. Age modified the MetS-FI association in ages ≥ 85, such that it was stronger at higher ages (p-interaction = 0·01). Low BMI, high BMI, and metabolic syndrome were associated with higher physiological age, contributing to overall health status among older individuals and potentially accelerating aging.

**Supplementary information:**

The online version contains supplementary material available at 10.1007/s11357-023-01032-9.

## Introduction

High body mass index (BMI) is among the leading risk factors for disability and mortality [1], incurring a substantial economic burden worldwide [2]. Obesity, defined as BMI ≥ 30 kg/m^2^, often co-occurs with other cardiometabolic risk factors, including hypertension, hyperglycemia, and dyslipidemia, used to diagnose metabolic syndrome (MetS) [3]. The presence of high BMI and these cardiometabolic components, independently or simultaneously as MetS, increase the risk of various late-life diseases such as type II diabetes (T2D) and cardiovascular diseases [4–6]. The epidemic proportions of obesity and MetS and their associations with adverse late-life health pose a pressing global health challenge in an increasingly aging population [1, 2].

The aging process is characterized by a gradual decline in physiological function that precipitates increased susceptibility to morbidity and mortality [7]. Nonetheless, there is substantial heterogeneity in aging, which chronological age fails to capture. Measures of physiological age, including the frailty index (FI) and the functional aging index (FAI), aim to quantify this heterogeneity in aging [8–11]. FI is a widely accepted multidimensional measure of health in aging, calculated as the proportion of health deficits relative to the total number of deficits considered [9, 10]. However, as FI measures the accumulation of health deficits that are often chronic, it typically increases over time and may not reflect health improvement. In contrast, FAI, a newer physiological age measure, quantifies current functional capacity and may better measure the fluctuations in functional abilities and health [11]. Therefore, while correlated, FI and FAI encompass distinct aspects of physiological aging and are complementary health measures [11, 12].

Obesity shares pathophysiological hallmarks with aging, such as chronic inflammation, insulin resistance, mitochondrial dysfunction, and metabolic dysregulation, and midlife obesity has been suggested to potentially increase the risk of late-life diseases by accelerating the aging process [13, 14]. Moreover, there is mounting evidence linking obesity to increased frailty risk [15], substantiating the association between obesity and accelerated physiological aging.

Metabolic health is also linked to aging [7] and frailty [16, 17]. However, despite the close relationship between BMI and MetS, previous research primarily evaluated them independently in relation to frailty [18, 19]. It is thus unclear how BMI and MetS interactively associate with physiological aging, and examining these associations may deepen our understanding of the aging process.

Therefore, this study examined how longitudinal measures of BMI and MetS jointly associate with physiological aging, measured by FI and FAI, and whether these associations change with chronological age.

## Methods

### Study population

**Fig. 1:**
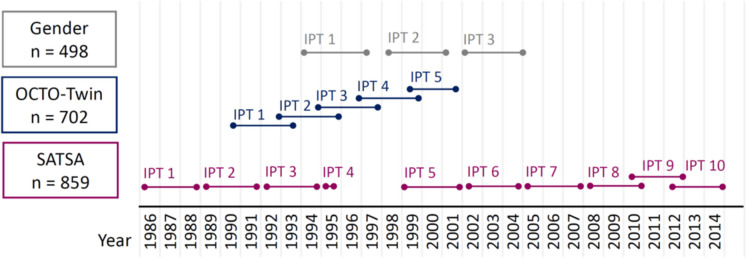
Timeline of in-person-testing occasions in GENDER, OCTO-Twin, and SATSA. This timeline presents when in-person testing occurred in the cohort studies GENDER, OCTO-Twin, and SATSA. Abbreviations: IPT – in-person-testing, n – sample size

The study drew data from the three longitudinal sub-studies of aging within the Swedish Twin Registry, a population-based register of virtually all twins born in Sweden, with repeated physical measures and questionnaire data (Fig. 1). GENDER [20] is comprised of 498 opposite-sex twins born between 1906 and 1925 who participated in up to three waves of in-person testings (IPT) between 1995 and 2006. OCTO-Twin [21] is comprised of 702 same-sex twins aged 80 and above (born between 1893 and 1913) who participated in up to five IPT waves between 1991 and 2001. SATSA [22] includes 859 same-sex twins over the age of 50 (born between 1900 and 1948) who participated in up to 10 IPT waves between 1986 and 2014. The IPT waves were conducted relatively similarly in all three studies, and data could, therefore, be pooled across studies, resulting in a sample size of 2,059 individuals who participated in at least one IPT (Supplementary Fig. S1). All participants provided informed consent, and the current study received approval from the Swedish Ethical Review Authority (2022-06634-01).

### Exposures

Trained nurses measured the participants’ height and weight at each IPT, and BMI was calculated as weight in kilograms per square of height in meters (kg/m^2^). We defined MetS based on modified NCEP-ATP III criteria [6], excluding the measure of central adiposity due to its strong correlation with BMI (Pearson correlation coefficient = 0·8 in our data). Therefore, MetS was defined as the presence of two or more (≥ 2) of the following cardiometabolic risk factors: hypertension, hyperglycemia, low high-density lipoprotein cholesterol (HDL), and hypertriglyceridemia, using measured biomarkers and self-reported disease diagnoses and medication use (Table 1). Systolic and diastolic blood pressure were measured with participants seated in GENDER, and supine in OCTO-Twin and SATSA, after five minutes of rest and a second reading after a one-minute pause. The lower of the two readings was recorded. Glucose, high-density lipoprotein cholesterol (HDL), and triglyceride levels were measured from venous blood serum collected under fasting or non-fasting conditions. Venous blood glucose levels were not measured in GENDER and OCTO-Twin, and triglyceride levels were not measured in OCTO-Twin. Self-reported medication use, and disease information were gathered in each IPT. Self-reported medication use was considered when ascertaining hyperglycemia, hypertriglyceridemia, and low HDL, and self-reported T2D diagnosis when ascertaining hyperglycemia.

**Table 1:** Criteria and thresholds used for ascertaining cardiometabolic risk factors included in the defination of metabolic syndrome

Cardiometabolic risk factors	Diagnostic criteria	GENDER	OCTO-Twin	SATSA
Hypertension	Systolic BP ≥ 135 mmHg	X	X	X
	Diastolic BP ≥ 85 mmHg	X	X	X
Hyperglycemia	Fasting BG ≥ 6‧1 mmol/L			X
	Non-fasting BG ≥ 7‧0 mmol/L			X
	Self-reported use of diabetic medications	X	X	X
	Self-reported diagnosis of type II diabetes	X	X	X
Low HDL	< 1‧03 mmol/L in males	X*	X ^┼^	X
	< 1‧30 mmol/L in females	X*	X ^┼^	X
	Self-reported use of lipid-lowering medications	X	X	X
Hypertriglyceridemia	Fasting TG ≥ 1‧70 mmol/L	X^ǂ^		X
	Non-fasting TG ≥ 2‧1 mmol/L	X^ǂ^		X
	Self-reported use of lipid-lowering medications	X	X	X

An ‘x’ in the cell represents the criteria used to ascertain the presence of cardiometabolic risk factors. Each cardiometabolic risk factor was deemed present when any one of the diagnostic criteria was fulfilled. * indicates measures available only in IPT3 and ^ǂ^ indicates measures available in IPT1 and IPT3 in GENDER. ^┼^ indicates measures available only in IPT2 in OCTO-Twin. Metabolic syndrome was defined as the presence of at least 2 (≥ 2) of the above four cardiometabolic risk factors. Abbreviations: BG – venous blood glucose levels, BP – blood pressure, HDL – high-density lipoproteins cholesterol from venous blood sampling, TG – triglyceride level from venous blood sampling

### Outcomes

#### Frailty index

FI is the percentage of deficits present out of a total number of deficiencies considered based on Rockwood’s model of accumulation of health deficits [23]. A higher FI indicates a higher physiological age. The health deficits considered included self-reported symptoms, diseases, and disabilities. In prior work, Bai and colleagues [24] calculated FI for the same material; we applied the same method but excluded self-reported T2D diagnoses from the calculation to avoid overlap with the MetS, leaving 41 health deficits in GENDER and SATSA and 40 in OCTO-Twin (Supplementary Table S1). As in the previous study, participants with > 20% missing health deficits necessary to compute FI were excluded, while missing items were imputed for those with ≤ 20% missing data [24]. FI was multiplied by 100 to represent the percentage of deficits.

#### Functional aging index

FAI is a composite score derived from gait speed, grip strength, and peak expiratory flow (PEF) that were measured by trained nurses and self-reported subjective sensory ability [11]. A higher FAI score indicates a higher physiological age. As described elsewhere [11], gait speed was the time to walk 3 m and back; grip strength was the highest of three measures from each hand taken using dynamometers or vigorimeters; and PEF was the better of two trials measured with portable spirometers. Before calculating FAI, grip strength was regression-corrected for sex, and PEF was corrected for body volume by dividing it by the individual’s squared height in meters. FAI was standardized to a mean of 50 and a standard deviation of 10.

### Covariates

Age in years was included as a time-varying covariate, while sex, education, and smoking history were measured at baseline and constant in all models. Sex was categorized as female or male at birth. Education was dichotomized into < 7, or ≥ 7 years, corresponding to compulsory or more than compulsory education, respectively, for these birth cohorts. Smoking history was categorized as ever or never smoked.

### Statistical analysis

The statistical workflow is detailed in the Supplementary methods and illustrated in Supplementary Figure S2. Briefly, both outcomes (FI and FAI) were modeled separately as a function of BMI and MetS measured at the same IPT wave. We used linear mixed-effects models with random intercepts at the twin-pair level and random intercepts and linear age effects at the individual level to account for correlations within twin-pairs and individuals. Age, smoking history, education, and sex were included as fixed effects in all models. Restricted cubic splines (RCS) were used to model curvilinear associations of age (Supplementary Fig. S3 and S4) and BMI with the outcomes. The goodness of fit was compared via likelihood ratio tests (LRT) for nested models or Akaike’s information criterion (AIC) for general models.

We assessed three-way effect modifications for the combination of BMI, MetS, and age to examine if the joint association of BMI and MetS with physiological age (FI or FAI) changed with chronological age. We found evidence supporting a better fit for the three-way model for FI [*p*-value of interaction from LRT (p-interaction) = 0·006], but not FAI (p-interaction = 0·56). Consequently, we fitted age-stratified models for FI, but not FAI.

#### Age-stratified models of FI

We stratified FI analyses by age categories: less than 65 years (< 65), 65 to < 85 years (65–85), and 85 years and above (≥ 85), where the functional relationship with FI in the lowest and highest age category was mostly linear based on the graphical presentation of predicted FI over age (Supplementary Fig. S3). In addition, we re-evaluated the linearity of age and BMI within each age stratum with RCS. Next, we re-examined three-way effect modifications for BMI, MetS, and age within each age stratum. When a significant three-way interaction was present, we compared it with simpler models, which included combinations of two-way interactions between age, BMI, and MetS, and the main effect models to select the most parsimonious final model. *P*-values ≤ 0·05 were considered statistically significant.

##### Sensitivity analyses

We conducted sensitivity analyses to assess the stability of the results from the final models and the contributions from individual metabolic health components by:

- including sub-study (SATSA, GENDER, OCTO-Twin) as a covariate to adjust for potential between-study heterogeneity;
- including the year of exposure measurement in 10-year intervals (1985 – < 1995, 1995 – < 2005, 2005 – < 2015) as a covariate to assess the influence from changes in incidence and prevalence of obesity and MetS across time periods on our results;
- including waist-hip ratio (WHR), a measure of central adiposity, in the ascertainment of MetS; high central adiposity was defined as WHR ≥ 0·90 in males and ≥ 0·85 in females, and MetS was defined as having ≥ 3 cardiometabolic risk factors;
- separately include hypertension, hyperglycemia, hypertriglyceridemia, and low HDL, instead of MetS, to assess their specific contribution to physiological aging.

Stata version 17·0 was used to perform the statistical analyses, and R package ggplot2 was used to generate plots.

## Results

From 2,059 possible participants, 1,825 individuals (6,052 measures) were eligible in the analyses of FI, and 1,691 individuals (5,257 measures) in the analyses of FAI, after excluding individuals with > 1 missing metabolic health measure or without BMI, FI, or FAI measurements (Supplementary Fig. S1). In the FI sample, 355 individuals (806 measures) had measures taken at age < 65, 1,591 individuals (3,928 measures) at age 65–85, and 619 individuals (1,318 measures) at age ≥ 85. Table 2 describes the characteristics of all participants at their first measurement for FI and FAI, and at their first measurement within each age stratum for FI.

**Table 2:** Descriptive statistics of participants from the first in-person testing in the full sample for FI and FAI, and stratified by age category for FI

	FI analysis								FAI analysis	
Sample type	Full sample		< 65		65 – 85		≥ 85		Full sample	
n of participants	1,825		355		1,591		619		1,691	
n of measures	6,052		806		3,928		1,318		5,257	
Females, n (%)	1,077	(59·0)	203	(57·2)	911	(57·3)	425	(68·7)	982	(58·1)
Age in years, mean (SD)	73·6	(9·93)	57·2	(4·70)	74·6	(5·95)	86·5	(1·40)	73·0	(9·87)
BMI in kg/m^2^, mean (SD)	25·5	(4·01)	26·0	(4·04)	25·9	(4·05)	24·6	(3·93)	25·6	(3·96)
FI in %, mean (SD)	14·5	(10·11)	7·7	(5·95)	14·2	(9·77)	20·9	(11·31)	13·9	(9·59)
FAI, mean (SD)	48·9	(11·62)	42·0	(8·67)	48·9	(11·11)	57·6	(12·05)	49·3	(11·89)
Education ≥ 7 years, n (%)	807	(44·2)	204	(57·5)	728	(45·8)	226	(36·5)	773	(45·7)
Ever-smokers, n (%)	892	(48·9)	206	(58·0)	797	(50·1)	246	(39·7)	841	(49·7)
High blood pressure, n (%)	1,631	(89·7)	267	(75·2)	1,408	(91·7)	477	(84·7)	1,502	(89·1)
Hyperglycemia, n (%)	171	(9·4)	23	(6·5)	181	(11·4)	87	(14·1)	154	(9·1)
HTG, n (%)	306	(16·8)	67	(18·9)	330	(20·8)	58	(9·4)	299	(17·7)
Low HDL, n (%)	45	(2·5)	26	(7·3)	101	(6·3)	87	(14·1)	57	(3·4)
MetS, n (%)	414	(22·68)	78	(22·0)	436	(27·4)	140	(22·6)	399	(23·6)
n metabolic abnorm., mean (SD)	1·2	(0·64)	1·1	(0·78)	1·3	(0·70)	1·1	(0·75)	1·2	(0·66)

Descriptive statistics were taken from the baseline measures of the full sample for FI and FAI, and the analytical sample for FI within age stratum. Continuous variables are presented as mean and standard deviation (SD), while categorical variables are in numbers (n) and percentages (%). Abbreviations: abnorm – abnormalities, BMI – body mass index, FAI – functional aging index, FI – frailty index, HDL-C – high-density lipoprotein cholesterol, HTG – hypertriglyceridemia, MetS – metabolic syndrome, n – number, SD – standard deviation

### BMI and MetS in relation to FI

**Fig. 2:**
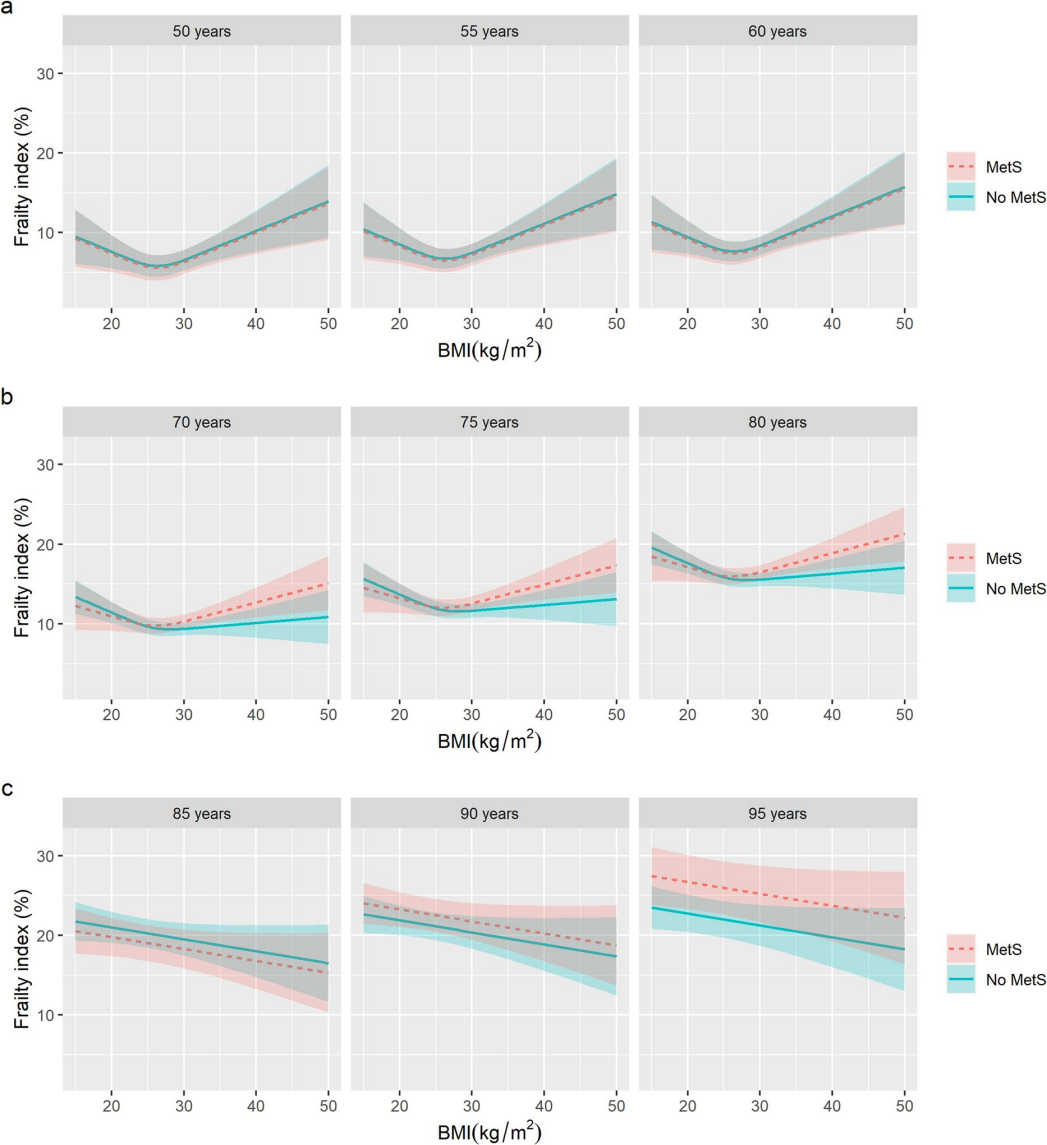
Association between BMI and physiological age measured by the frailty index, stratified by metabolic syndrome and age stratum. Predicted values of frailty index derived from mixed-effects models with random effects on the intercept on the twin pair level and the intercept and linear age on the individual level, and sex, education, and smoking history were included as fixed effects. Models were fitted separately for age groups **a**. < 65, **b**. 65–85, and **c**. ≥ 85, and three representative ages were plotted in each stratum. BMI were modeled as restricted cubic splines with 3 knots, except at age ≥ 85 where it was included as a linear term. Age were modeled as linear terms, except at ages 65-85 where it was a restricted cubic spline with 3 knots. Blue solid lines and corresponding confidence intervals represent predicted values for individuals without metabolic syndrome, and red dashed lines and corresponding confidence intervals for individuals with metabolic syndrome. Abbreviations: BMI – body mass index, MetS – metabolic syndrome

Figure 2 illustrates the expected FI as a function of BMI and MetS. To assess the relative contribution of age to the models, we plot the expected FI at three different representative ages in each stratum.

In the < 65 age group, we found no statistically significant evidence for effect modification (all p-interactions > 0·33), supporting a simple additive model for BMI and MetS (Supplementary Table S2). The association between BMI and FI was statistically significant (Wald test *p*-value = 2·5E-3) and non-linear (Wald test *p*-value = 1·3E-3). As seen in Fig. 2a, the association between BMI and FI was U-shaped, with the lowest expected FI at a BMI of 26·3 kg/m^2^ and increasing from there for both lower and higher BMI values. The difference in expected FI between participants with and without MetS was small and not statistically significant (β_MetS_ = -0·24, 95% CI = -1·12–0·64, *p*-value = 0·59). Age in this stratum had minor impact on FI curves, affecting only their offset on the vertical (FI) axis (Fig. 2a).

For measurements collected in ages 65–85, a statistically significant interaction modified the joint association between BMI and MetS with FI (p-interaction = 0·02). Similar to the < 65 age group, the association between BMI and FI was statistically significant (Wald test *p*-value = 3·0E-05), non-linear (Wald test *p*-value = 2·2E-03), and U-shaped (Fig. 2b, Supplementary Table S3). However, due to the interaction between BMI and MetS, the expected FI over BMI differed for participants with and without MetS. The BMI with the lowest FI was slightly higher in the MetS group (28·1 kg/m^2^) than in the non-MetS group (26·0 kg/m^2^), but up to BMI 28 kg/m^2^, the expected FI was relatively similar for both groups. Above BMI 28 kg/m^2^, there was a stronger linear increase in FI in the MetS group, compared to a very modest linear increase in the non-MetS group, suggesting a stronger association between BMI and physiological age among participants with obesity and MetS, compared to participants with obesity but without MetS. Like the < 65 age stratum, the FI curves were consistent across ages, but had a more pronounced shift along the vertical axis (Fig. 2b).

In the ≥ 85 age group, we found a statistically significant interaction between age (as a linear term) and MetS in their joint association with FI (p-interaction = 0·01), such that the expected FI increased by an additional 0·52% (95% CI = 0·11–0·93) with every year of age in the MetS group, compared to the non-MetS group (Supplementary Table S4). We observed a linear inverse association, albeit not statistically significant, between BMI and FI (β_BMI_ = -0·15, 95%CI = -0·33–0·03, *p*-value = 0·09); see Fig. 2c. Compared to earlier age groups, this stratum shows comparatively slight FI changes across ages in the non-MetS group but substantial changes in the MetS group due to the effect modification (Fig. 2c).

### BMI and MetS in relation to FAI

**Fig. 3:**
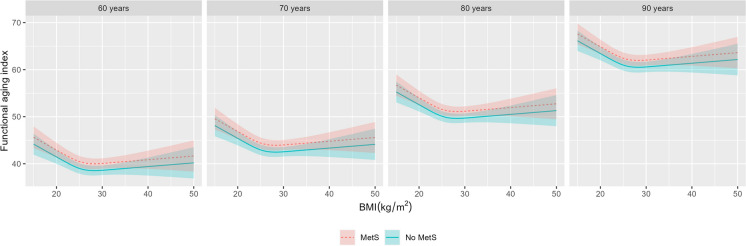
Association between BMI and physiological age measured by the functional aging index, stratified by metabolic syndrome across ages. Predicted values of functional aging index (FAI) derived from a mixed-effects model with random effects on the intercept on the twin pair level and the intercept and linear age on the individual level, and sex, education, and smoking history were included as fixed effects. Age and BMI were specified as restricted cubic splines with 3 knots. Blue solid lines and corresponding confidence intervals represent predicted values for individuals without metabolic syndrome, and red dashed lines and corresponding confidence intervals for individuals with metabolic syndrome. Each curve shows the BMI-FAI association at 4 representative ages, 60 to 90 years, at 10-year intervals. Abbreviations: BMI – body mass index, MetS – metabolic syndrome

Unlike for FI, there was no evidence of effect modification (all p-interactions > 0·11) when FAI was the outcome, supporting a simple additive model for BMI and MetS across ages. The fitted model for FAI is visually represented by plotting the expected FAI as a function of BMI and MetS at different representative ages (Fig. 3).

In the additive model, the association between BMI and FAI was statistically significant (Wald test *p*-value = 3·1E-7) and non-linear (Wald test *p*-value = 1·4E-4). The association between BMI and FAI was U-shaped, with the lowest expected FAI at a BMI of 28·4 kg/m^2^, and increasing for both lower and higher BMI values from there. Notably, the U-shape of the FAI curve was not symmetrical around the lowest point, but showed a stronger linear increase towards lower BMI, and a more modest linear increase for higher BMI.

Participants with MetS had, on average, a higher FAI than participants without MetS (β_MetS_ = 1·46, 95% CI = 0·94–1·97, *p*-value = 2·9E-9, Supplementary Table S5) regardless of age or BMI.

### Sensitivity analyses

The results remained robust after further adjusting the final models for the sub-study (Supplementary Table S6), time period (Supplementary Table S7), and including WHR in the definition of MetS (Supplementary Table S8).

**Fig. 4:**
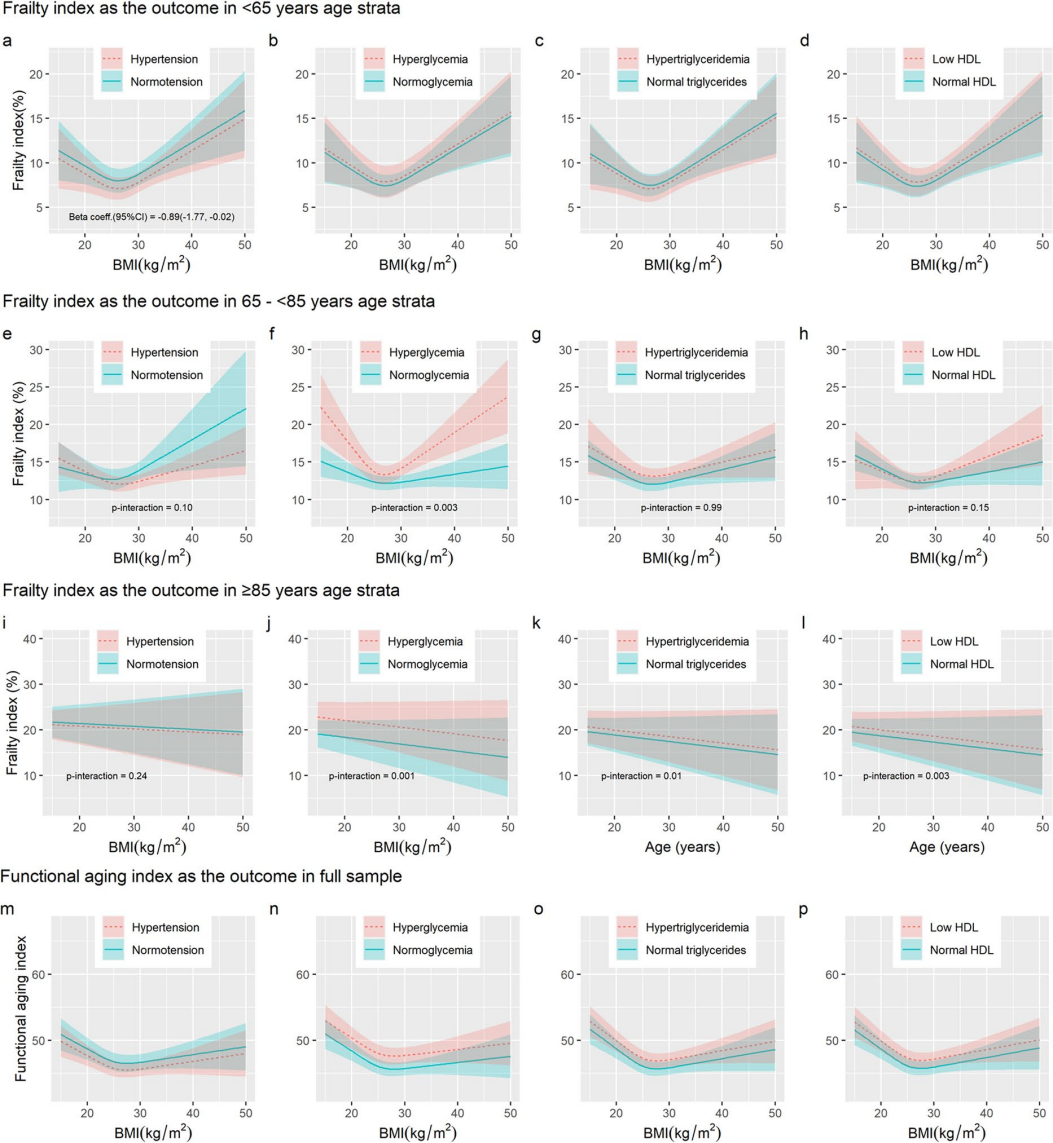
Association of BMI with physiological age measured by frailty index and functional aging index by the status of individual cardiometabolic risk factors. Predicted values were derived from mixed-effects model with random effects on the intercept on the twin pair level and on the intercept and linear age on the individual level, and age, sex, education, and smoking history were included as fixed effects. Panels **a** – **d** depict associations with FI in ages < 65 years at reference age 59 years. Panels **e** – **h** depict FI- ages 65–85 years at reference age 76 years, and **i** – **l** depict associations with FI at reference age 88 years. Panels **m** – **p** show FAI-BMI at reference age 75 years. BMI was specified as restricted cubic splines (RCS) with 3 knots for panels **a** – **l**, and linear for panels **m** – **p**. Age was specified as linear term for panels **a** – **d** and **i** – **l**, RCS for the remaining panels. P-interaction is the *p*-value obtained from likelihood ratio tests by comparing a model with interaction with an additive model. Blue continuous lines are predicted values for individuals without the cardiometabolic risk factor, and the blue area around it indicates the confidence intervals. Red dotted lines are predicted values for individuals with the cardiometabolic risk factor, and the red area around it indicates the confidence intervals. Abbreviations: BMI – body mass index, HDL – high-density lipoprotein cholesterol

Models testing individual components of MetS are presented in Fig. 4 and Supplementary Table S9. For each individual MetS component, the U-shaped association between BMI and FI remained in ages ≥ 65 years, or FAI across ages, and the BMI-FI association in ages ≥ 85 remained non-significant.

In ages < 65, hypertension (HTN) was associated with lower FI (β_HTN_ = -0.89, 95% CI = -1‧77,-0‧02, *p*-value = 0.05), while all other MetS components, like MetS itself, were not associated with FI (Fig. 4a – d).

In ages 65–85, hyperglycemia had the strongest association with higher FI. A statistically significant interaction modified the association between BMI and hyperglycemia with FI (p-interaction = 0·003), and the BMI with the lowest FI in those with hyperglycemia and normoglycemia were 26.7 kg/m^2^ and 27.4 kg/m^2^, respectively. Those with hyperglycemia(HG) showed a much stronger linear increase toward lower and higher BMIs when compared to those with normoglycemia (Fig. 4f). The interactions between BMI and other MetS components were not statistically significant (Fig. 4e, g, and h). The main effect of hypertriglyceridemia (HTG) was associated with higher FI (β_HTG_ = 1.09, 95% CI = 0.24,1.94, *p*-value = 0.01), but the main effects of hypertension and low HDL were not statistically significant.

In ages ≥ 85, there were significant interactions between age and hyperglycemia (p-interaction = 0.001), HTG (p-interaction = 0.01), and low HDL (p-interaction = 0.003), but not hypertension (Fig. 4i – l). In the presence of hyperglycemia, hypertriglyceridemia, and low HDL, the expected FI increased by an additional 0·83% (95% CI = 0·32–1.34), 0·87% (95% CI = 0·20–1.53), and 0·73% (95% CI = 0·25–1.21), respectively, with every year of age.

For FAI, hyperglycemia (β_HG_ = 3‧27, 95% CI = 2‧09,4‧45, *p*-value = 7.1E-7), hypertriglyceridemia (β_HTG_ = 1.21, 95% CI = 0.63,1.79, *p*-value = 4.3E-5) and low HDL (β_HDL_ = 1‧17, 95% CI = 0‧56,1‧78, *p*-value = 2.0E-4) were associated with higher FAI (Fig. 4m – p). In contrast, hypertension was associated with lower FAI (β_HTN_ = -1.03, 95% CI = -1‧71,-0‧34, *p*-value = 0.003).

## Discussion

In summary, we found a U-shaped association between BMI and physiological age, where underweight and obesity were associated with the highest physiological age, except in those aged ≥ 85 years, where the association between BMI and FI was not statistically significant, but showed a trend indicating a lower physiological age with higher BMI levels. MetS was associated with higher physiological age, except with FI in ages < 65. Additionally, MetS modified the BMI-FI association at age 65–85, such that individuals with MetS had higher physiological age at higher BMI levels than individuals without MetS. In ages ≥ 85 years, age modified the MetS-FI association such that the association was stronger at higher ages. Notably, the sample sizes for analyses of FI at ages < 65 and ≥ 85 were limited, cautioning the interpretation of associations specific to these groups.

Although this study, to the best of our knowledge, is the first to examine how BMI and MetS jointly associate with physiological aging, our findings align with previous studies examining the association of BMI and MetS separately with frailty. Studies on BMI and frailty risk have reported a similar U-shaped relationship [15], reiterating the well-established association between excessively low or high BMI levels and adverse late-life health outcomes [25, 26]. In the current study, MetS was associated with higher physiological age measured by FAI across ages and FI in ages ≥ 65 years, even after adjusting for BMI, agreeing with previous meta-analyses demonstrating an association between MetS and increased frailty risk [16, 27].

### Age-specific effects

Some studies have reported higher BMI in late life associating with better health outcomes, a much-debated phenomenon known as the obesity paradox [28]. Such paradoxical association may arise from increased risk for chronic illnesses at older ages, leading to unintentional weight loss related to pre-existing disease processes, side effects of their treatments, or sarcopenia [29]. Our study did not find this inverse association between BMI and physiological age. Our study did not replicate this overall inverse association between BMI and physiological age – the closest we found was a negative association between BMI and FI after age 85, which failed to reach statistical significance. Nonetheless, two features that appeared consistently in our models before age 85 may lead to the impression of such an inverse association in some study designs: low BMI was consistently associated with higher physiological age, and the lowest physiological age was observed within the overweight range. Consequently, studies that did not have enough observation in the obesity range or did not properly account for age effects may have failed to capture the strong and positive association between BMI and physiological age across the obesity range that we have found in our material.

Alternatively, previous findings reflecting an obesity paradox may be biased by loss of information by categorizing BMI broadly or assuming BMI as a linear measure. By treating BMI as a continuous variable and carefully considering non-linearity in its associations, we identified a U-shaped BMI-physiological aging association, except for FI, at ages ≥ 85.

Age moderated the association of BMI and MetS with FI, but not FAI. Although both FI and FAI attempt to measure physiological aging, they differ conceptually and empirically. FI is an accumulative measure of health deficits that generally increases over time, whereas FAI measures current functional capacity, which can fluctuate with health. While both measures generally increase with age, it is plausible that the health deficits included in FI are more susceptible to age-specific associations with BMI and MetS than the functional measures included in FAI.

### Metabolic heterogeneity of the BMI phenotypes

MetS modified BMI-FI association in ages 65–85. The strength of association between a higher BMI in the overweight to obesity range and higher FI was greater in individuals with MetS than those without MetS, indicating that the simultaneous manifestation of high BMI and MetS may increase physiological aging, as measured by FI, more than high BMI or MetS alone.

By examining the individual MetS components separately, hyperglycemia showed the strongest association with higher physiological age and may be a major contributor to the MetS-physiological age association. Conversely, hypertension was associated with lower physiological age, aligning with previous research on hypertension and health outcomes [30]. Biases from reverse causality and selective survival may explain such inverse associations. However, these findings using individual cardiometabolic components should be interpreted cautiously due to variations in data collection and availability across the different sub-studies, which led to diverse combinations of data used to determine the presence of each cardiometabolic component. Additionally, the classification of hypertension did not consider medication use, while the classification of hyperglycemia, hypertriglyceridemia, and low HDL did. Future research jointly examining BMI and individual cardiometabolic risk factors could render a more comprehensive understanding of their roles in physiological aging.

### Biological underpinnings of obesity, metabolic syndrome, and physiological aging

Obesity and aging share many pathophysiological characteristics, and it has been suggested that obesity may accelerate the aging process, explaining the link between obesity and age-related diseases [7, 13, 14]. At the molecular level, obesity and aging are both characterized by oxidative stress driven by chronic inflammation and biological oxidations and changes such as mitochondrial dysfunction, cellular senescence, and telomere shortening [13]. At the organ level, obesity and aging are both associated with adipose tissue dysfunction, expressed as insulin resistance [13].

The prevalence of MetS increases with age, and deregulated nutrient-sensing is one of the twelve hallmarks of aging [7]. MetS and other metabolic diseases are also linked to other hallmarks of aging, including mitochondrial dysfunction [7, 13], and it is noteworthy that the twelve hallmarks of aging are closely integrated. Our results, where a high BMI in the obesity range and MetS were associated with increased physiological aging, aligned with the hypothesis that obesity accelerates aging. Nonetheless, it should be noted, especially given the biological similarities, that the associations may also stem from other factors jointly influencing BMI, metabolic health, and physiological aging.

This study leveraged rich, longitudinal data to examine how BMI and MetS associate with physiological aging while considering differences in associations across aging. Data collection within GENDER, OCTO-Twin, and SATSA were conducted relatively similarly, involving comprehensive and repeated questionnaires, health assessments such as weight, height, and blood pressure measurements, and venous blood sampling; pooling these three sub-studies of aging enabled us to maximize the sample size. By including GENDER and OCTO-Twin, it allowed oversampling of late adulthood, ages that are typically underrepresented in aging studies. Additionally, our study was based on data that comprised BMI measured by health care professionals, FI from self-reports, and FAI and MetS status from a combination of self-reports and objective measures. Furthermore, we examined two complementary physiological age measures, FI and FAI, capturing different facets of physiological aging.

However, the sample sizes in the < 65 years and ≥ 85 age strata for FI analyses were small, potentially resulting in sparse data, especially in the extreme BMI levels. While pooling data from the three cohorts provides better power, participants entered the sub-studies at different ages and time periods. There were also variations in the ascertainment of cardiometabolic components based on data availability of the respective sub-studies (Table 1). For instance, OCTO-Twin, which did not measure triglyceride levels, only used lipid-lowering medication to indicate hypertriglyceridemia. OCTO-Twin and GENDER only used T2D diagnosis and medication use to ascertain hyperglycemia. However, using MetS as a collective measure of several metabolic health factors may be more robust to differences in individual components. Moreover, sensitivity analyses with further adjustment for sub-study and time period suggest neither the variations between sub-studies nor changes in incidence and prevalence of obesity and MetS across time period affect the findings in this study.

In conclusion, a BMI in the obesity range and MetS were associated with higher physiological aging, in line with the hypothesis that obesity and MetS accelerate the aging process [13]. A low BMI was also associated with higher physiological age and may be a warning sign of ill health. Considering a low or high BMI, and metabolic health in tandem as signs of advanced physiological aging may better facilitate the assessments of overall health status among older individuals.

### Supplementary information

Below is the link to the electronic supplementary material.

Supplementary file1 (PDF 694 KB)

## Data Availability

The data involved in this study were obtained from the Swedish Twin Registry (STR). STR is an international resource that can be applied for access at https://ki.se/en/research/swedish-twin-registry-for-researchers.
