# Supplementary material for: Interplay of body mass index and metabolic syndrome: association with physiological age from midlife to late-life
Source: GeroScience. 2023 Dec 16;46(2):2605–17. doi: 10.1007/s11357-023-01032-9 (PMC10828240; doi:10.1007/s11357-023-01032-9)
Supplement: Supplementary file 1 — Supplementary file1 (PDF 694 KB) [file 11357_2023_1032_MOESM1_ESM.pdf]

Supplementary materials

Interplay of body mass index and metabolic syndrome: association with physiological age from midlife to late life

Peggy Ler, M.Sc.[1], Alexander Ploner, Ph.D.[1], Deborah Finkel, Ph.D.[2,3], Chandra A. Reynolds, Ph.D.[4], Yiqiang Zhan, Ph.D.[5], Juulia Jylhävä, Ph.D.[1,6], Anna K. Dahl Aslan, Ph.D.[7], Ida K. Karlsson, Ph.D.[1]

[1] Karolinska Institutet, Department of Medical Epidemiology and Biostatistics, Stockholm, Sweden
[2] University of Southern California, Center for Economic and Social Research, California, USA
[3] Jönköping University, Institute of Gerontology, Jönköping, Sweden
[4] University of Colorado Boulder, Institute for Behavioral Genetics, Colorado, USA.
[5] Sun Yat-Sen University, School of Public Health, Shenzhen Campus, Guandong, China
[6] University of Tampere, Faculty of Social Sciences, Unit of Health Sciences and Gerontology Research Center, Tampere, Finland
[7] University of Skövde, School of Health Sciences, Skövde, Sweden

Corresponding author's name, email:
Peggy Ler, peggy.ler@ki.se

Contents

Supplementary methods: Description of the statistical workflow…...2

Table S1: Self-reported items included in the derivation of the frailty index..4

Table S2: Estimated model parameters for the joint association of BMI and metabolic syndrome with frailty index for participants aged <65 years..6

Table S3: Estimated model parameters for the joint association of BMI and metabolic syndrome with frailty index for participants aged 65 to <85 years..7

Table S4: Estimated model parameters for the joint association of BMI and metabolic syndrome with frailty index for participants aged 85 years and above 8

Table S5: Estimated model parameters for the joint association of BMI and metabolic syndrome with functional aging index 9

Table S6: Estimated model parameters for the joint association of BMI and metabolic syndrome with frailty index and functional aging index, additionally adjusted for study..10

Table S7: Estimated model parameters for the joint association of BMI and metabolic syndrome with frailty index and functional aging index, additionally adjusted for period..11

Table S8: Estimated model parameters for the joint association of BMI and metabolic syndrome, with waist-hip-ratio included as a criterion, with frailty index and functional aging index..12

Table S9: Estimated model parameters for the joint association of BMI and individual components of metabolic syndrome with frailty index and functional aging index..13

Figure S1: Data flow chart..15

Figure S2: Statistical workflow..16

Figure S3: Curvilinear association between frailty index and age as restricted cubic splines..........................17

Figure S4: Curvilinear association between functional aging index and age as restricted cubic spline..................18

Reference..19

Supplementary Methods: Description of the statistical workflow

The modeling flow is summarized in Figure S2 (p16) in this supplementary materials. Linear mixed-effects models were applied throughout, with random effects on the intercept on the twin-pair level (to adjust for the relatedness among twins pairs) and on the intercept and linear age on the individual level. Age, smoking history, education, and sex were included as fixed effects.

In step 1, we examined non-linearity of change in FI and FAI across aging, by modeling the marginal association between age and outcomes FI and FAI separately without BMI and MetS in the model. Restricted cubic splines (RCS) were used to model curvilinear associations between age and the outcomes. The knots of restricted cubic splines were placed at percentiles per Harrel's[1] recommendations, where with three knots, knots were placed at the 10th, 50th, and 90th percentile, with 4 knots at the 5th, 35th, $65^{th,}$ and 95th percentile and with 5 knots at 5th, 27.5^{th}, 50th, 72.5^{th} and 95th percentile. Likelihood ratio tests (LRT) of nested models were applied to obtain p-values for non-linearity (p-linearity) by comparing a model with the variable as RCS to one with the same variable as a linear term. We found statistically significant non-linearity in the association between age and both outcomes. We arrived at a final shape for FI as a function of age as RCS with 5 knots (p-linearity < 1e-4) and for FAI as a function of age as an RCS with 3 knots (p-linearity < 1e-4) (Supplementary Figure 4 – 5).

In step 2, we tested for non-linearity in the association between BMI and FI or FAI by modeling the marginal association between BMI and the outcomes, with age as a spline term identified in the previous steps and MetS as a covariate. We found statistically significant evidence for non-linearity in BMI by comparing models with a linear BMI term and RCS with three knots for FI and FAI (p-linearity < 1e-4).

In step 3, we tested if chronological age modifies the joint associations of BMI and MetS with the outcomes, by including a three-way effect modification for BMI, MetS, and age; as well as a two-way effect modification of the joint effects of BMI and MetS. These models contained all exposures, covariates, and spline terms for age and BMI identified in the previous steps. The significance of effect modifications (p-interaction) was tested using likelihood ratio tests (LRT), comparing the model with the interaction term to a nested model without the interaction. When a significant three-way interaction was present, we compared it with simpler models, which included interaction terms between 1) age and MetS, age and BMI, BMI, and MetS; 2) BMI and age, MetS and age; 3) BMI and age only; 4) MetS and age only; and 5) BMI and MetS only. As the final model, we selected the most parsimonious model, including only statistically significant interactions.

For FAI, neither including a three-way interaction between BMI, MetS, and age fit (p-interaction = 0·56) nor including a two-way interaction between BMI and MetS (p-interaction = 0·54) significantly improved model fit; therefore, we selected the additive model with no interactions as the final model. In this final model, BMI and age were RCSs with three knots, as mentioned above.

For FI, a model including an interaction between BMI, MetS, and age had a significantly better fit than a model without the three-way interaction term (p-interaction=1e-4), as well as the simpler models, indicating that the joint association between BMI and MetS with physiological age varies with chronological age.

Therefore, in step 4, we stratified the models for FI by age categories: less than 65 years (<65), 65 to <85 years (65–85), and 85 years and above (≥85) years, where the relationship in the lowest and highest age category appears mostly linear based on the graphical presentation of predicted FI over age derived from step 1 (Supplementary Figure S4). In addition, we reassessed the linearity of age within each age stratum. Age as a linear term best fits the data for models of ages <65 and ≥85 years. In ages 65-85 years, a model with three knots had the best fit. We also re-evaluated the linearity of BMI in each age stratum and found curvilinear associations between BMI and FI in the <65 (p-linearity = 0·002) and 65-85 age stratum (p-linearity = 1e-4) and thus kept BMI as RCS with three knots in these age strata. No evidence of a curvilinear association between BMI and FI was present in the ≥85 age stratum, and BMI was modeled as a linear term in this age group.

Finally, we repeated step three to assess the presence of three- and two-way effect modification of the joint effects of BMI, MetS, and age, and BMI and MetS, respectively, on FI in each age category, selecting the most parsimonious model with significant interactions. In the <65 age group, there was no evidence of three-way or two-way interactions between combinations of BMI, MetS, and age (p-interaction > 0·31). Therefore, we selected the additive

model with no interactions as the final model. In the 65-85 age group, models including the interaction between BMI and MetS improved model fit (p-interaction = 0·02). In the ≥85 age stratum, there was evidence of significant three-way interactions, when compared to the model without the interaction (p-interaction = 0·01). After comparing to simpler models, a model including an interaction between MetS and age was selected as the final model for the ≥85 age stratum.

Table S1: Self-reported items included in the derivation of the frailty index

Items	SATSA	OCTO-Twin	GENDER
General health status	X	X	X
Limited from doing things one would normally like to do due to health status	X	X	X
Cancer or leukemia	X		X
Rheumatoid arthritis	X	X	X
Arthritis	X	X	
Chronic bronchitis or emphysema	X	X	
Cataracts	X	X	X
Chest pain	X	X	X
Circulation problems in arms or legs	X	X	X
Persistent cough	X		
Goiter or other gland problems	X	X	X
Heart failure	X	X	X
Hypertension	X	X	X
Kidney disease	X	X	X
Osteoporosis	X	X	X
Sciatica	X	X	X
Anemia	X	X	
Cerebral hemorrhage or blood clot in brain	X	X	
Dizziness	X	X	
Gastric ulcer	X	X	X
Allergies/allergic manifestations	X	X	
Asthma	X	X	
Showering and bathing	X	X	X
Getting in and out of bed	X		X
Dressing and undressing	X		X
Self-grooming	X		
Walking	X	X	X
Trouble getting to the toilet in time	X	X	X
Traveling further distances	X		X
Housework	X		X
Preparing meals	X		

**Supplementary Table 2 continued**			
**Items**	**SATSA**	**OCTO-Twin**	**GENDER**
Managing medications	X		X
Managing money	X		
Using telephone	X		X
Grocery shopping	X		X
Hearing acuity	X	X	
Vision acuity	X	X	
Feeling lonely the past week	X	X	X
Feeling depressed the past week	X		X
Feeling happy the past week	X		
Feeling tired the past week	X		
Keeping body fit		X	
Heart attack		X	X
Vascular spasm in leg		X	X
Herpes		X	
Migraine		X	X
Glaucoma		X	X
Speech impairment		X	X
Eczema		X	X
Hip joint impairment		X	X
Neck pain		X	
Shoulder pain		X	
Gall bladder		X	X
Insomnia		X	X
Psychological problems		X	
Stroke			X
Epilepsy			X
Liver disease			X
Gout			X
Picking something up from the floor			X
Handling small things with your fingers			X
**Total number of items**	**41**	**40**	**41**

An ‘x’ in the cell of the table represents the presence of the item in the calculation of FI.

Table S2: Estimated model parameters for the joint association of BMI and metabolic syndrome with frailty index for participants aged <65 years

	β	95% Confidence intervals	
		Lower limit	Upper limit
BMI spline 1 (^{knots}22 , 26)	0·37	-0·19	0·93
BMI spline 2 (^{knots}26 , 32)	-0·84	-1·35	-0·33
Metabolic syndrome	-0·24	-1·12	0·64
Age	0·18	0·10	0·26
Female	1·62	0·26	2·99
Ever-smoker	1·31	0·05	2·58
Education > 7years	0·22	-0·98	1·42
Constant	6·33	4·59	8·08
Random Effects			
SD (Intercept within pairs)	2·31	1·45	3·67
SD (Slope within individuals)	0·30	0·21	0·43
SD (Intercept within individuals)	4·25	3·65	4·95
Correlation (within individuals)	0·62	0·16	1·08
SD(Residuals)	3·52	3·25	3·81

Estimates derived from mixed-effects model with random effects on the intercept on the twin pair level and on the intercept and linear age on the individual level, and age, sex, education, and smoking history included as fixed effects, for 355 participants aged <65 years with 806 measures. Age was specified as a linear term centered at 59 years and BMI as restricted cubic splines with 3 knots (denoted by ^{knots}). Abbreviations: β – beta-coefficients, BMI – body mass index, SD – standard deviation.

Table S3: Estimated model parameters for the joint association of BMI and metabolic syndrome with frailty index for participants aged 65 to <85 years

	β	95% Confidence Intervals	
		Lower limit	Upper limit
BMI spline 1 (^{knots}21 , 26)	-0·48	-0·85	-0·11
BMI spline 2 (^{knots}26 , 31)	-0·50	-0·83	-0·18
Metabolic syndrome	0·34	-0·35	1·04
MetS x BMI spline 1	0·61	0·15	1·06
MetS x BMI spline 2	-0·04	-0·49	0·40
Age spline 1 (^{knots}68, 77)	3·73	3·47	3·99
Age spline 2 (^{knots}77, 84)	-0·79	-0·96	-0·61
Female	2·41	1·54	3·29
Ever-smoker	1·15	0·29	2·01
Education > 7years	-0·66	-1·46	0·13
Constant	12·33	11·26	13·40
Random Effects			
SD (Intercept within pairs)	3·31	2·69	4·07
SD (Slope within individuals)	0·49	0·44	0·56
SD (Intercept within individuals)	6·61	6·19	7·06
Correlation (Within individuals)	0·77	0·61	0·92
SD(Residuals)	3·97	3·83	4·11

Estimates derived from a mixed-effects model with random effects on the intercept on the twin pair level and on the intercept and linear age on the individual level, and age, sex, education, and smoking history included as fixed effects, for 1,591 participants aged 65 to <85 years with 3,928 measures. Age and BMI were specified as restricted cubic splines with 3 knots (denoted by ^{knots}). An interaction term between BMI and MetS was included in this model. Abbreviations: β – beta-coefficients, BMI – body mass index, MetS –metabolic syndrome, x – in interaction, SD – standard deviation

Table S4: Estimated model parameters for the joint association of BMI and metabolic syndrome with frailty index for participants aged 85 years and above

	β	95% Confidence Intervals	
		Lower limit	Upper limit
BMI	-0·15	-0·33	0·03
Metabolic syndrome	0·34	-0·87	1·55
Age	0·18	-0·04	0·39
MetS x age	0·52	0·11	0·93
Female	4·41	2·33	6·49
Ever-smoker	2·53	0·56	4·50
Education > 7years	-1·39	-3·16	0·38
Constant	18·13	15·90	20·37
Random Effects			
SD (Intercept within pairs)	3·74	2·07	6·75
SD (Slope within individuals)	1·16	0·93	1·44
SD (Intercept within individuals)	8·94	7·95	10·06
Correlation (within individuals)	-0·03	-0·24	0·17
SD (Residuals)	4·85	4·53	5·18

Estimates derived from a mixed-effects model with random effects on the intercept on the twin pair level and on the intercept and linear age on the individual level, and age, sex, education, and smoking history included as fixed effects, for 619 participants aged ≥85 years with 1,318 measures. BMI and age were specified as linear terms centered at 22.5kg/m^2 and 88 years, respectively. An interaction term between MetS and age was included in this model. Abbreviations: β – beta-coefficients, BMI – body mass index, MetS – metabolic syndrome, x – interaction, SD – standard deviation.

Table S5: Estimated model parameters for the joint association of BMI and metabolic syndrome with functional aging index

	β	95% Confidence Intervals	
		Lower limit	Upper limit
BMI spline 1 (^{knots}21 , 25)	-0·69	-1·07	-0·31
BMI spline 2 (^{knots}25 , 31)	-0·67	-0·99	-0·34
Metabolic syndrome	1·46	0·94	1·97
Age Spline 1 (^{knots}62, 77)	6·55	6·21	6·89
Age Spline 2 (^{knots}77, 87)	-1·68	-1·94	-1·42
Female	3·70	2·66	4·73
Ever-smoker	1·22	0·23	2·21
Education > 7years	-3·09	-4·01	-2·17
Constant	47·91	46·67	49·15
Random Effects			
SD (Intercept within pairs)	4·94	4·32	5·65
SD (Slope within individuals)	0·30	0·26	0·35
SD (Intercept within individuals)	6·87	6·41	7·38
Correlation (within individuals)	0·84	0·63	1·04
SD(Residuals)	5·86	5·71	6·01

Estimates derived from a mixed-effects model with random effects on the intercept on the twin pair level and on the intercept and linear age on the individual level, and age, sex, education, and smoking history included as fixed effects, for 1,691 participants with 5,257 observations. Age and BMI were specified as restricted cubic splines with 3 knots. ^{knots} denotes the location of knots for restricted cubic splines. Abbreviations: β – beta-coefficients, BMI – body mass index, SD – standard deviation

Table S6: Estimated model parameters for the joint association of BMI and metabolic syndrome with frailty index and functional aging index, additionally adjusted for study

Outcomes	FI				FAI	
Age strata	65 – <85 years		≥85 years		Full sample	
	β	(95% CI)	β	(95% CI)	β	(95% CI)
BMI spline 1	-0·42	(-0·79,-0·04)			-0·77	(-1·14,-0·40)
BMI spline 2	-0·49	(-0·82,-0·17)			-0·68	(-1·00,-0·37)
BMI			-0·12	(-0·30, 0·05)		
MetS	0·56	(-0·14,1·26)	0·68	(-0·55,1·91)	1·07	(0·55,1·58)
MetS x BMI spline 1	0·57	(0·12,1·03)				
MetS x BMI spline 2	-0·06	(-0·51,0·38)				
Age spline 1	3·35	(3·06,3·64)			7·50	(7·12,7·88)
Age spline 2	-0·77	(-0·95,-0·59)			-1·59	(-1·86,-1·33)
MetS x age			0·48	(0·07,0·89)		
Age			0·19	(-0·03,0·40)		
Female	2·44	(1·57,3·30)	4·6	(2·52,6·68)	3·42	(2·44,4·40)
Ever-smoker	1·20	(0·35,2·06)	2·51	(0·56,4·46)	1·10	(0·16,2·05)
Education > 7years	-0·51	(-1·30,0·29)	-1·20	(-2·98,0·58)	-3·24	(-4·12,-2·35)
Study (Reference GENDER)						
OCTO-Twin	2·21	(0·87,3·55)	-0·19	(-3·78,3·39)	0·34	(-1·11,1·79)
SATSA	-1·48	(-2·47,-0·49)	-3·09	(-6·90,0·73)	7·27	(6·10,8·44)
Constant	12·35	(11·13,13·57)	18·71	(14·72,22·68)	44·77	(43·37,46·17)
Random effects						
SD (Intercept within pairs)	3·20	(2·59,3·96)	3·69	(2·05,6·67)	3·98	(3·34,4·73)
SD (Slope within individuals)	0·50	(0·44,0·56)	1·16	(0·93,1·44)	0·30	(0·25,0·35)
SD (Intercept within individuals)	6·50	(6·08,6·95)	8·90	(7·92,10·00)	6·81	(6·36,7·30)
Correlation (within individuals)	0·72	(0·57,0·87)	0·02	(-0·19,0·23)	0·77	(0·58,0·96)
SD (Residuals)	3·97	(3·84,4·11)	4·85	(4·53,5·29)	5·85	(5·70,6·00)

Estimates derived from mixed-effects models with random effects on the intercept on the twin pair level and on the intercept and linear age on the individual level, and age, sex, education, smoking history, and study included as fixed effects. BMI was specified as a linear term and centered at 22·5, and age was specified as a linear term and centered at 88 in the ≥85 years age strata. BMI and age were specified as restricted cubic splines with 3 knots otherwise. Sensitivity analysis was not performed in the <65 years age strata since all participants were from the SATSA study. Abbreviations: β – beta-coefficients, BMI – body mass index, FAI – functional aging index, FI – frailty index, MetS – metabolic syndrome, x – in interaction, SD – standard deviation

Table S7: Estimated model parameters for the joint association of BMI and metabolic syndrome with frailty index and functional aging index, additionally adjusted for time period

**Outcomes**	**FI**						**FAI**	
**Age strata**	**<65 years**		**65 – <85 years**		**≥85 years**		**Full sample**	
	**β**	**(95% CI)**	**β**	**(95% CI)**	**β**	**(95% CI)**	**β**	**(95% CI)**
BMI spline 1	0.45	(-0.12, 1.02)	-0.39	(-0.76, -0.02)			-0.67	(-1.05, -0.29)
BMI spline 2	-0.85	(-1.37, -0.32)	-0.48	(-0.81, -0.16)			-0.68	(-0.96, -0.36)
BMI					-0.16	(-0.33, 0.01)		
MetS	-0.11	(-1.04, 0.82)	0.75	(0.05, 1.45)	0.32	(-0.83, 1.48)		
MetS x BMI spline 1			0.43	(-0.02, 0.87)				
MetS x BMI spline 2			-0.26	(-0.70, 0.18)				
Age spline 1			3.83	(3.56, 4.09)			6.51	(6.09, 6.94)
Age spline 2			-0.83	(-1.01, -0.66)			-1.73	(-1.99, -1.47)
MetS x age					0.46	(0.08, 0.84)		
Age	0.17	(0.06, 0.28)			0.28	(0.06, 0.49)		
Female	1.86	(0.47, 3.25)	2.42	(1.55, 3.29)	4.21	(2.18, 6.24)	3.68	(2.65, 4.70)
Ever-smoker	1.51	(0.22, 2.80)	1.22	(0.36, 2.08)	2.71	(0.80, 4.63)	1.23	(0.25, 2.22)
Education > 7years	0.32	(-0.93, 1.56)	-0.35	(-1.16, 0.45)	-1.51	(-3.27, 0.25)	-3.07	(-3.99, -2.14)
Period (Reference 1985 – <1995)								
1995 – <2005	-0.42	(-1.51, 0.67)	-0.93	(-1.46, -0.40)	-0.57	(-1.66, 0.51)	-0.74	(-1.30, -0.19)
2005 – <2015	0.61	(-1.12, 2.34)	-2.00	(-2.80, -1.19)	-0.004	(-2.22, 2.21)	0.39	(-0.50, 1.27)
Constant	5.96	(4.17, 7.76)	12.75	(11.66, 13.84)	18.63	(2.98, 6.59)	48.14	(46.90, 49.39)
Random effects								
SD (Intercept within pairs)	0.30	(1.48, 3.70)	3.22	(2.60, 4.00)	4.43	(2.98, 6.59)	4.82	(4.20, 5.54)
SD (Slope within individuals)	4.22	(0.21, 0.44)	0.47	(0.42, 0.54)	1.03	(0.82, 1.30)	0.30	(0.26, 0.35)
SD (Intercept within individuals)	0.56	(3.61, 4.94)	6.66	(6.24, 7.11)	8.79	(7.86, 9.83)	6.85	(6.38, 7.35)
Correlation (Within individuals)	3.53	(0.11, 1.01)	0.77	(0.62, 0.93)	0.02	(-0.19, 0.23)	0.83	(0.63, 1.03)
SD (Residuals)	0.30	(3.25, 3.83)	3.89	(3.76, 4.03)	4.83	(4.53, 5.14)	5.86	(5.71, 6.01)

Estimates derived from mixed-effects models with random effects on the intercept on the twin pair level and on the intercept and linear age on the individual level, and age, sex, education, smoking history, and period included as fixed effects. BMI was specified as a linear term and centered at 22·5 in the ≥85 years age strata. Age was specified as a linear term and centered at 59 and 88 in the <65 and ≥85 years age strata, respectively. BMI and age were specified as restricted cubic splines with 3 knots otherwise. Abbreviations: β – beta-coefficients, BMI – body mass index, FAI – functional aging index, FI – frailty index, MetS – metabolic syndrome, x – in interaction, SD – standard deviation

Table S8: Estimated model parameters for the joint association of BMI and metabolic syndrome, with waist-hip-ratio included as a criterion, with frailty index and functional aging index

**Outcomes**	**FI**						**FAI**	
**Age strata**	**<65 years**		**65 – <85 years**		**≥85 years**		**Full sample**	
	**β**	**(95% CI)**	**β**	**(95% CI)**	**β**	**(95% CI)**	**β**	**(95% CI)**
BMI spline 1	0·39	(-0·18,0·95)	-0·41	(-0·78,-0·05)			-0·71	(-1·09,-0·33)
BMI spline 2	-0·83	(-1·34,-0·32)	-0·47	(-0·79,-0·15)			-0·67	(-0·99,-0·35)
BMI					-0·15	(-0·33,0·03)		
MetS	-0·38	(-1·33,0·57)	0·69	(-0·12,1·50)	-0·03	(-1·34,1·28)	1·56	(1·02,2·10)
MetS x BMI spline 1			0·38	(-0·13,0·88)				
MetS x BMI spline 2			-0·24	(-0·73,0·25)				
Age spline 1			3·72	(3·46,3·98)			6·55	(6·21,6·89)
Age spline 2			-0·79	(-0·97,-0·61)			-1·68	(-1·94,-1·42)
MetS x age					0·53	(0·06, 0·99)		
Age	0·18	(0·10,0·26)		(1·59,3·34)	0·19	(-0·02,0·40)		
Female	1·59	(0·22,2·96)	2·46	(0·30,2·03)	4·37	(2·28,6·45)	3·79	(2·75,4·82)
Ever-smoker	1·32	(0·05,2·59)	1·16	(-1·47,0·13)	2·53	(0·56,4·50)	1·21	(0·22,2·20)
Education > 7years	0·22	(-0·98,1·43)	-0·67	(11·20,13·33)	-1·40	(-3·18,0·37)	-3·09	(-4·01,-2·17)
Constant	6·35	(4·61,8·09)	12·26	(1·59,3·34)	18·25	(16·02,20·48)	47·93	(46·69,49·17)
Random effects								
SD (Intercept within pairs)	2·31	(1·46,3·67)	3·27	(2·65,4·04)	3·75	(2·08,6·75)	4·94	(4·32,5·65)
SD (Slope within individuals)	0·30	(0·21,0·43)	0·49	(0·44,0·56)	1·14	(0·92,1·42)	0·30	(0·26,0·35)
SD (Intercept within individuals)	4·25	(3·66,4·95)	6·62	(6·20,7·07)	8·94	(7·95,10·06)	6·89	(6·42,7·39)
Correlation (Within individuals)	0·61	(0·16,1·07)	0·76	(0·61,0·92)	-0·04	(-0·25,0·17)	0·83	(0·63,1·04)
SD (Residuals)	3·51	(3·24,3·80)	3·97	(3·84,4·11)	4·86	(4·55,5·20)	5·85	(5·71,6·00)

Estimates derived from mixed-effects models with random effects on the intercept on the twin pair level and on the intercept and linear age on the individual level, and age, sex, education, and smoking history included as fixed effects. BMI was specified as a linear term and centered at 22·5 in the ≥85 years age strata. Age was specified as a linear term and centered at 59 and 88 in the <65 and ≥85 years age strata, respectively. BMI and age were specified as restricted cubic splines with 3 knots otherwise. MetS was defined as having >2 components, including waist-hip ratio. Abbreviations: Physio. – physiological, BMI – body mass index, β – beta-coefficients, FI – frailty index, FAI – functional aging index, MetS – metabolic syndrome, SD - standard deviation, x – in interaction.

Table S9: Estimated model parameters for the joint association of BMI and individual components of metabolic syndrome with frailty index and functional aging index

Outcomes	FI						FAI	
Age strata	<65		65 - <85		≥85		Full sample	
	β	95% CI	β	95% CI	β	95% CI	β	95% CI
**Hypertension (HTN)**								
HTN	-0·89	(-1·77,-0·02)	-0·71	(-1·48,0·06)	-1·06	(-2·33,0·21)	-1·03	(-1·71,-0·34)
BMI spline 1	0·38	(-0·16,0·93)	0·66	(-0·10,1·43)			-0·42	(-0·82,-0·03)
BMI spline 2	-0·80	(-1·29,-0·30)	-0·65	(-1·31,0·01)			-0·59	(-0·93,-0·25)
BMI					-0·06	(-0·25,0·13)		
BMI spline 1 x HTN			-0·77	(-1·51,-0·03)				
BMI spline 2 x HTN			0·05	(-0·61,0·70)				
Age					-0·10	(-0·49,0·29)		
HTN x age					0·24	(-0·16,0·64)		
p-interaction			BMI x HTN: 0·10		HTN x age: 0·24			
**Hyperglycemia (HG)**								
HG	0·44	(-0·90,1·78)	2·1	(1·03,3·17)	2·04	(0·40,3·69)	3·27	(2·09,4·45)
BMI spline 1	0·33	(-0·22,0·88)	-0·24	(-0·59,0·11)			-0·53	(-0·92,-0·14)
BMI spline 2	-0·83	(-1·34,-0·32)	-0·43	(-0·74,-0·13)			-0·50	(-0·83,-0·16)
BMI					-0·15	(-0·32,0·03)		
BMI spline 1xHG			-0·16	(-0·80,0·49)				
BMI spline 2xHG			-1·08	(-1·70,-0·46)				
Age					0·15	(-0·06,0·36)		
HG x age					0·83	(0·32,1·34)		
p-interaction			BMI x HG: 0·003		HG x age: 0.001			
**Hypertriglyceridemia (HTG)**								
HTG	-0·42	(-1·30,0·47)	1·09	(0·24,1·94)	-0·66	(-2·87,1·54)	1·21	(0·63,1·79)
BMI spline 1	0·38	(-0·18,0·94)	-0·24	(-0·60,0·12)			-0·67	(-1·05,-0·29)
BMI spline 2	-0·82	(-1·33,-0·31)	-0·60	(-0·91,-0·28)			-0·68	(-1·00,-0·35)
BMI					-0·14	(-0·32,0·03)		
BMI spline 1xHTG			-0·04	(-0·56,0·48)				
BMI spline 2xHTG			-0·01	(-0·52,0·49)				
Age					0·20	(-0·003,0·41)		
HTG x age					0·87	(0·20,1·53)		
p-interaction			BMI x HTG: 0·99		HTG x age: 0·01			
**Low High-density lipoprotein-cholesterol levels (Low HDL)**								
Low HDL	0·48	(-0·61,1·57)	0·21	(-0·64,1·06)	-0·17	(-1·52,1·18)	1·17	(0·56,1·78)

BMI spline 1	0·33	(-0·22,0·88)	-0·30	(-0·65,0·05)			-0·62	(-1·00,-0·24)
BMI spline 2	-0·85	(-1·36,-0·34)	-0·53	(-0·84,-0·23)			-0·67	(-0·99,-0·35)
BMI					-0·14	(-0·32,0·03)		
BMI spline 1xHDL			0·45	(-0·08,0·99)				
BMI spline 2xHDL			-0·09	(-0·62,0·44)				
Age					0·19	(-0·02,0·39)		
HDL x age					0·73	(0·25,1·21)		
p-interaction			BMI x HDL: 0·15		HDL x age: 0·003			

Estimates derived from mixed-effects model with random effects on the intercept on the twin pair level and on the intercept and linear age on the individual level, and age, sex, education, and smoking history included as fixed effects. BMI was specified as a linear term and centered at 22·5 in the ≥85 years age strata. Age was specified as a linear term and centered at 59 and 88 in the <65 and ≥85 years age strata, respectively. BMI and age were specified as restricted cubic splines with 3 knots otherwise. P-interaction is the p-value obtained from likelihood ratio tests by comparing a model with interaction with an additive model. Abbreviations: β – beta-coefficients, BMI – body mass index, FAI – functional aging index, FI – frailty index, HDL – high-density lipoprotein cholesterol, HG – hyperglycemia, HTG – hypertriglyceridemia, HTN – hypertension, MetS – metabolic syndrome, SD - standard deviation, x – in interaction, p-interaction – p-value of interaction derived from likelihood ratio tests comparing a model with interactions with one without interactions.

Figure S1: Data flow chart

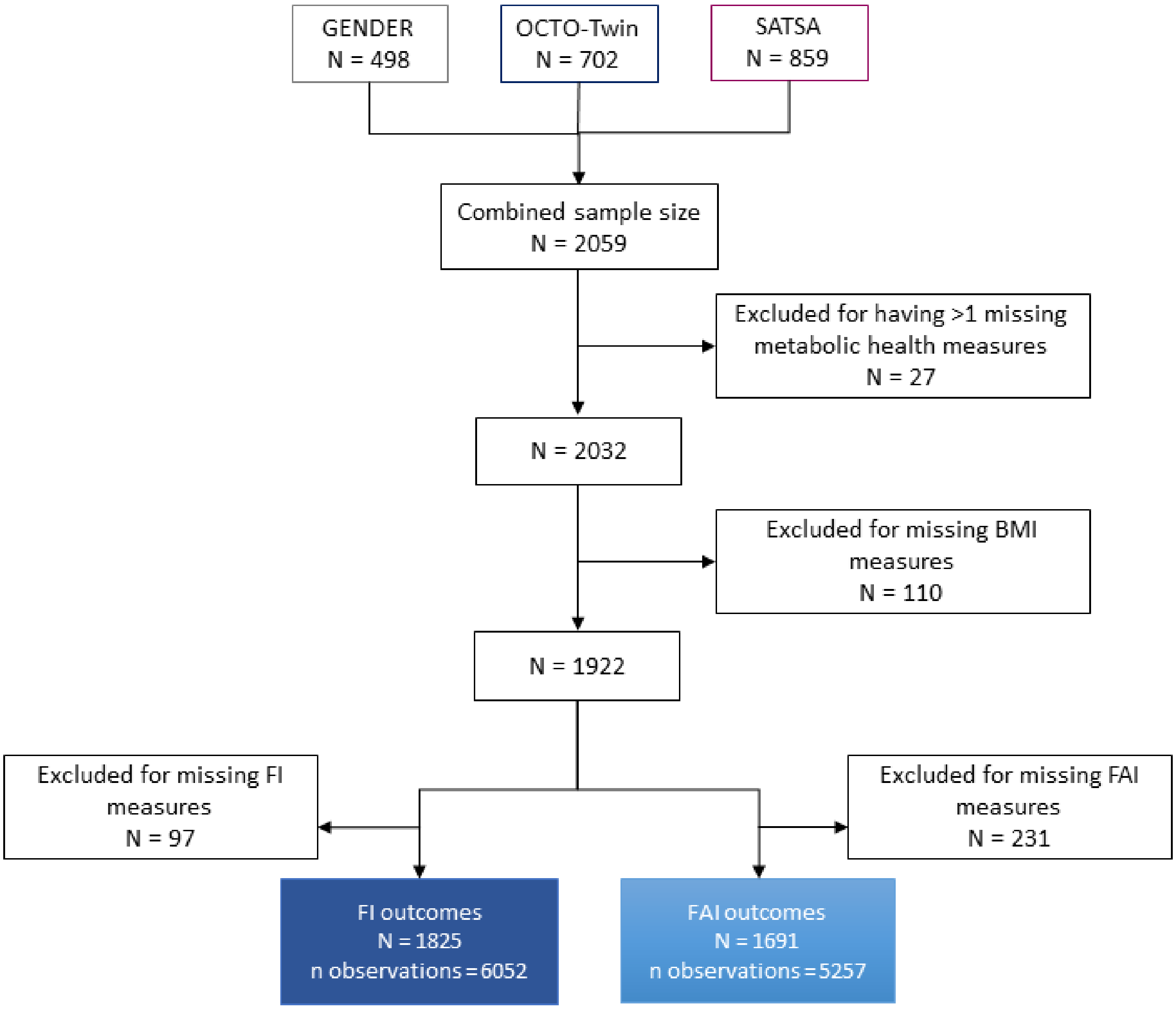

Abbreviations: FAI – functional aging index, FI – frailty index, N – number of individuals, n – number of observations.

Figure S2: Statistical workflow

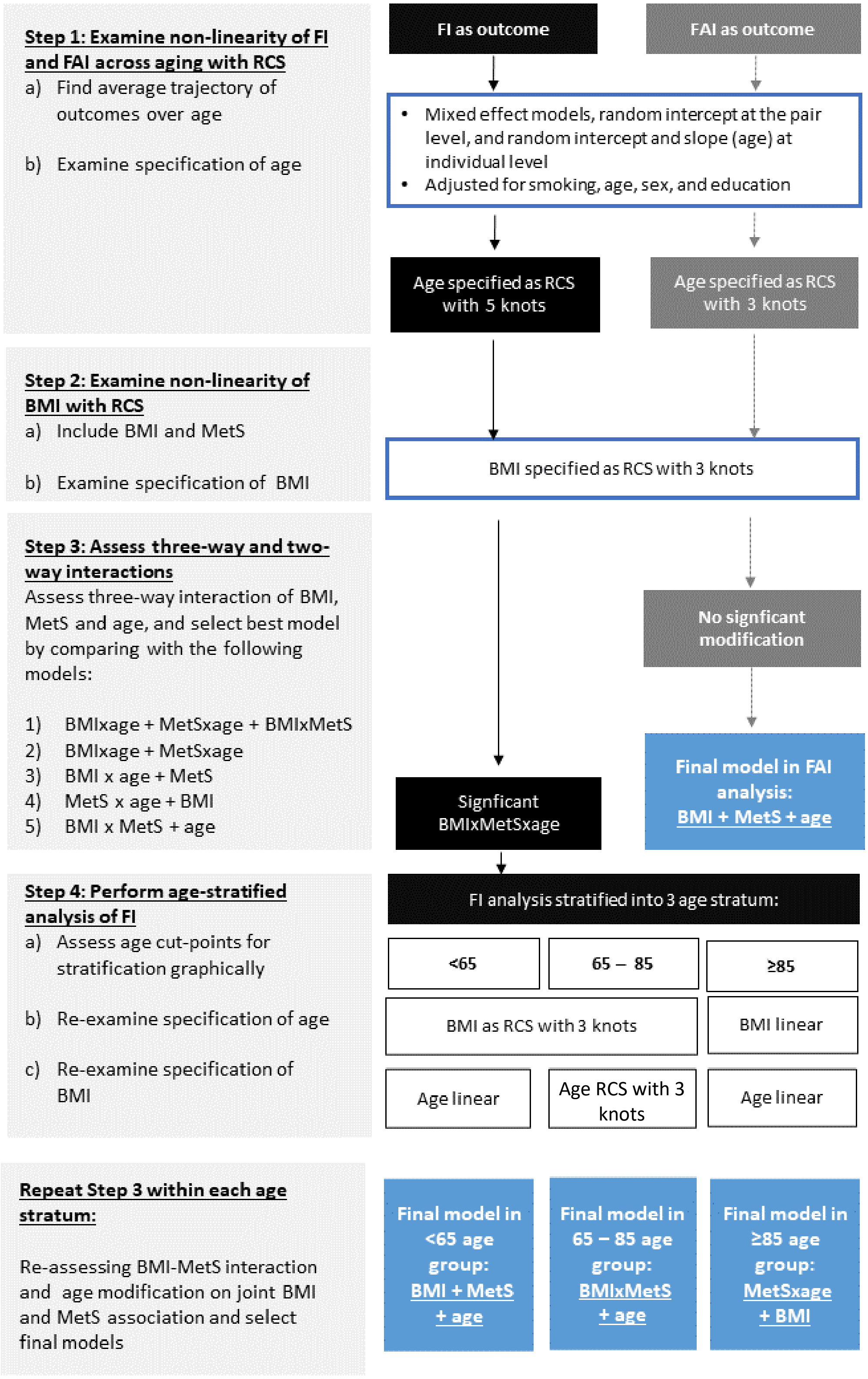

Abbreviations: BMI – body mass index, FAI – functional aging index, FI – frailty index, MetS – metabolic syndrome, RCS – restricted cubic splines, x - in interaction, + - additive

Figure S3: Curvilinear association between frailty index and age as restricted cubic splines.

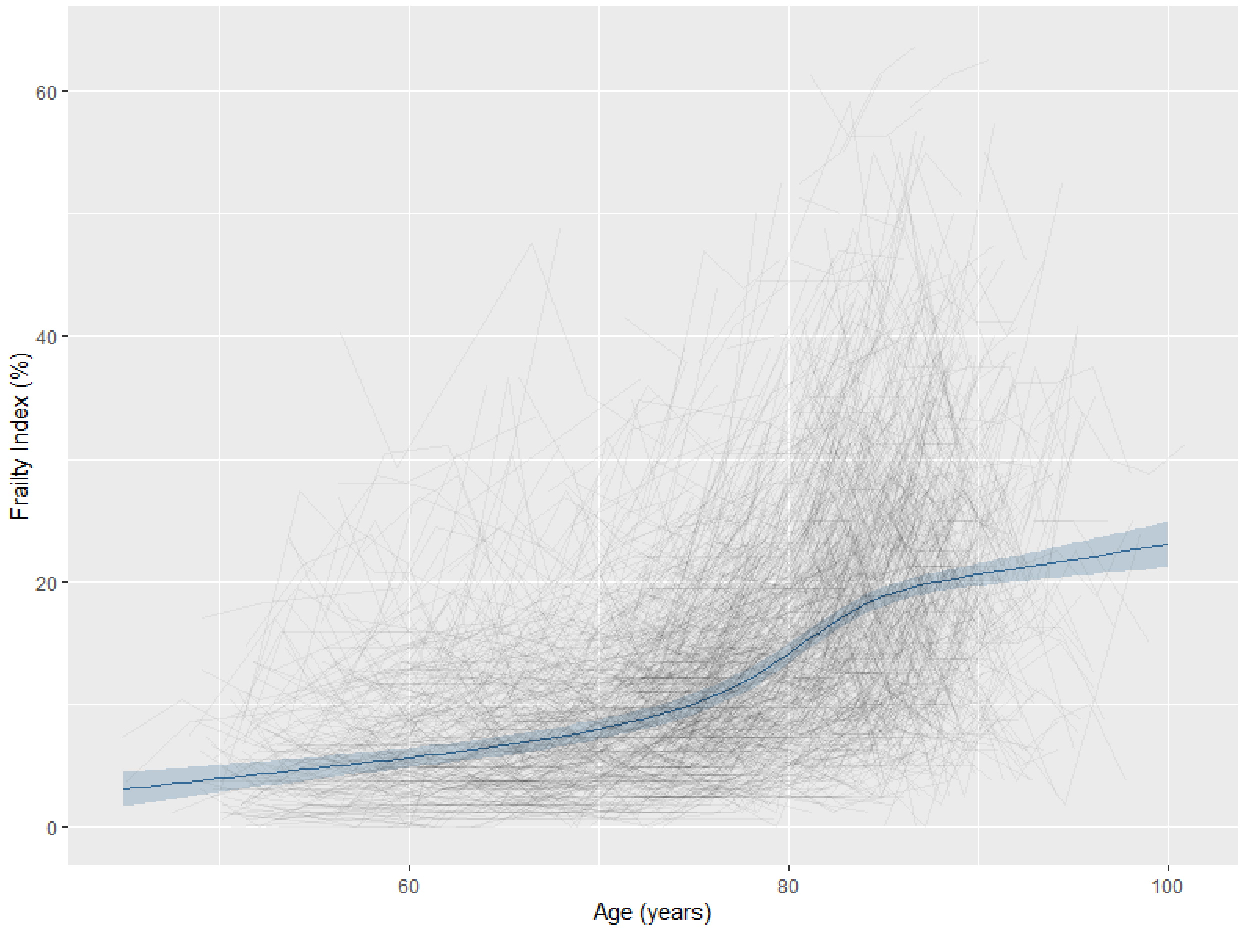

Predicted frailty index for all 1,825 participants, generated from a mixed-effects model with random effects on the intercept on the twin pair level and the intercept and linear age on the individual level, and age, sex, education, and smoking history included as fixed effects. Age was a restricted cubic spline with 5 knots. Each grey line depicts the change in frailty index for an individual over age. The dark blue line represents the predicted frailty index generated from the model, and the blue area around the line is the confidence intervals of the predictions.

Figure S4: Curvilinear association between functional aging index and age as restricted cubic spline

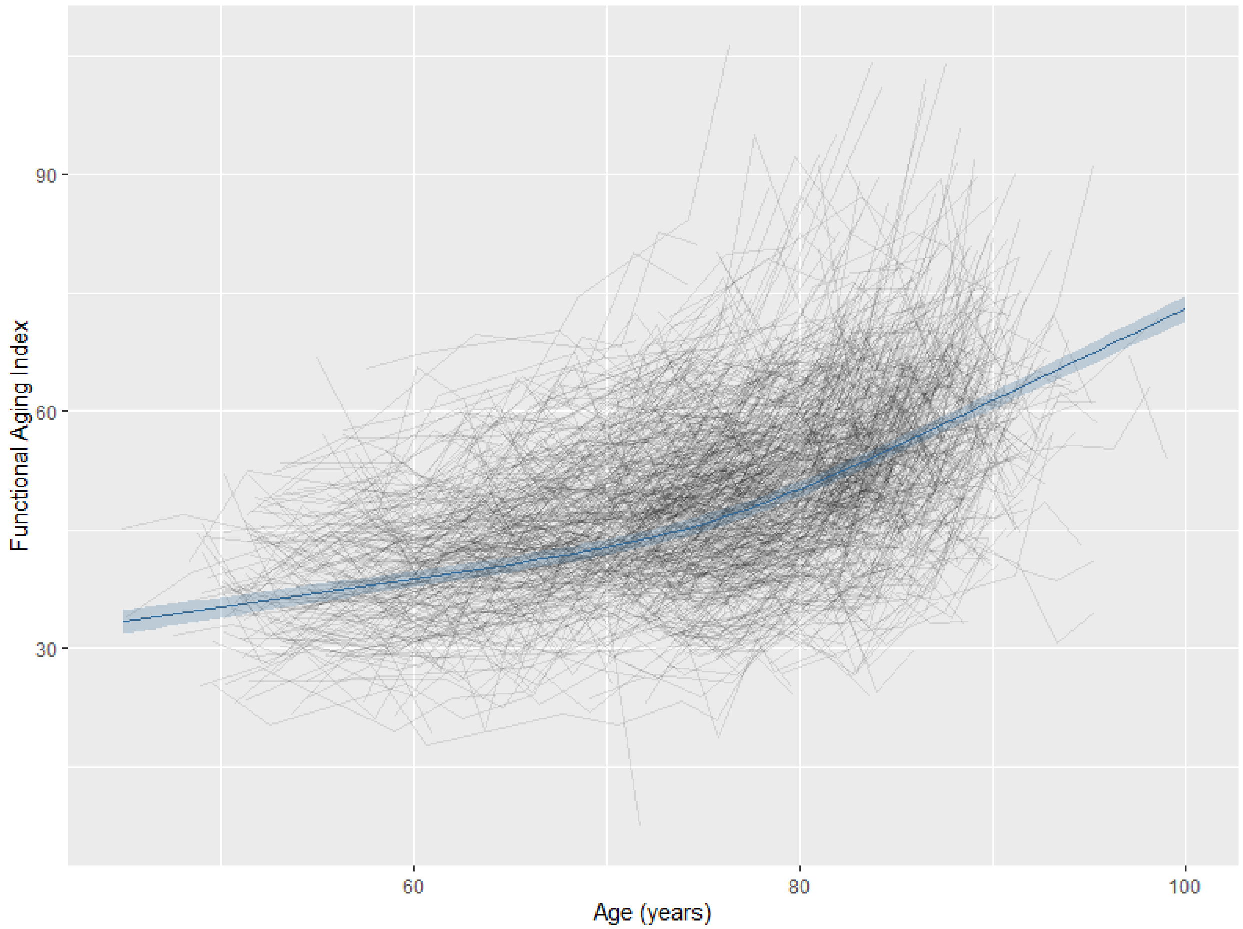

Predicted functional aging index in all 1,691 participants, generated from a mixed-effects modeling, with random effects on the intercept on the twin pair level and on the intercept and linear age on the individual level, and age, sex, education, and smoking history included as fixed effects. Age was a restricted cubic spline with 5 knots. Each grey line depicts the change in functional aging index for an individual over age. The dark blue line represents the predicted functional aging index generated from the model, and the area around the line is the confidence interval of the predictions.

Reference

1. Harrell FE. *Regression Modeling Strategies: With Applications to Linear Models, Logistic Regression, and Survival Analysis*. 1st 2001. ed. Springer series in statistics. Springer New York : Imprint: Springer; 2001.
